# Remarks on Hotz’s Operation for Entropion and Trichiasis, with Seven Cases

**Published:** 1882-06-20

**Authors:** A. G. Hobbs

**Affiliations:** Professor of Diseases of Eye, Ear and Throat in the Southern Medical College, Atlanta, Georgia


### Southern Medical Record:

EDITORS:

T. S. POWELL, M.D. W. T. GOLDSMITH, M.D. R. C. WORD, M.D.

*R. C. WORD, M.D., Managing Editor.*

☞ All Communications and Letters on Business connected with the RECORD must be addressed to the Managing Editor.

VOL. XII. ATLANTA, GA., JUNE 20, 1882. No. 6.

### ORIGINAL AND SELECTED ARTICLES.

### REMARKS ON HOTZ'S OPERATION FOR ENTROPION AND TRICHIASIS, WITH SEVEN CASES.

BY A. G. HOBBS, M. D.,

Professor of Diseases of Eye, Ear and Throat in the Southern Medical College, Atlanta, Georgia.

Read before the Georgia State Medical Association.

An entropion is a turning in of the free border of the eye-lid, with a consequent displacement of the ciliæ inwards—the latter condition being known as trichiasis.

Trichiasis may exist without entropion, by a simple displacement of the lashes inwards, but an entropion could not well exist without a trichiasis.

Entropion is most frequently the sequel of trachomatous conjunctivitis. The cicatricial contractions that result from trachoma draw upon the free margin of the lid, and this force, together with the contractions of the fibres of the orbicularis muscle, may with greater facility invert the lids of old people and debilitated persons with lax fibre and flabby skin, than those of young persons or robust individuals with firm and tense integument, though it is not infrequent that we meet with entropion even in these latter cases, where the trachomatous cicatrix is very firm and binding.

This incurvation of the tarsal cartilage is greatly facilitated by the diminished resistance of the cartilage (due to some infiltration in acute inflammation like trachoma). Upon this view have been founded the various methods of operation for the cure of entropion. Heretofore all these operations—though differing somewhat in detail—have been founded upon the common idea that it requires a strong traction power to bring an inverted eye-lid back to its normal position, and operators have naturally fallen into the error that by shortening the integument of the lid and producing a vertical cicatrix, that they could, in this manner, bring about sufficient traction force to pull the eye-lid back to its normal position. But experience has proven that even though the amount of integument taken from the lid be so great and the resultant cicatrix so strong that the lids cannot be closed, the entropion is not always remedied.

The question then is warranted, whether the idea of a strong traction is not altogether erroneous? or at least whether or not the traction has not been wrongly applied? In other words, whether the force is not wasted without affecting the free edge of the lid?

All the old operations have, then, been founded upon an error, as has been proven by the introduction of a new and more rational principle.

Dr. F. C. Hotz, of Chicago, was the first to describe this new principle to the profession, and since the introduction of this new operation, known by his name, it has become almost as universal for oculists of this country to resort to it as to Graefe's method of extracting hard cataracts.

In the language of Dr. Hotz, it is not difficult to demonstrate the erroneousness of the old operation, by a very few simple experiments: "Every oculist makes the experiment unconsciously when he wants to scrutinize the inside edge of a lid; and every physician may easily make it in order to convince himself of the correctness of the following observations: If you place a slightly bent probe upon the upper lid at the line of the upper border of the cartilage while the eye is closed, and then press the probe very slightly backwards and upwards, you will be astonished at the small amount of pressure it requires to overcome the worst degree of entropion. The inverted edge quickly and readily turns out as soon as the tension of the skin is slightly increased by the presure of the probe." If you compare this slight force with the amount of traction as represented by the width of the fold of skin which would have to be excised for the removal of the same degrees of inversion, the difference is quite obvious.

Then if these experiments be true, the tractive force that is necessary for the cure of an entropion is very small, if applied in the right direction.

A series of observations with reference to this point has lately proven that entropion is not in reality what it has always been thought to be, *i. e.*, a turning in of the free border of the cartilage proper, but it is nothing more nor less than a rolling in, or in other words, an inversion of the external integument; the skin near the edge is rolled in toward the conjunctiva, thereby giving the edge the peculiar rounded appearance, while the cartilage remains unchanged in shape and position.

Blepharospasm, which is an essential feature in the production of entropion, affects the integument rather than the tarsus. As soon as the spasm begins the skin is seen to move toward the free edge, and to be gathered into numerous horizontal folds.

This phenomenon, according to Stellwag, is due to the action of the orbicularis muscle, its fibres sandwiched between skin and tarsal cartilage and fascio-tarso-orbitalis run from one commissure to the other, those next the free edge are almost horizontal in course, the further they are removed from the margin the greater the curve of the arch they describe. When these arched fibres are shortened by contraction, their curves must become more or less straightened, because their origin and insertion are fixed and immovable points.

This change of curvature cannot be accomplished but by a change in position, *i. e.*, the middle portion of the muscle must make a decided movement toward the edge of the lid in order to approach as near as possible the straight line which is the shortest distance between the two commissures.

Now, this muscle is connected by cellular tissue with the skin and with the tarsal cartilage; the skin being movable, will readily follow the movements of the muscular fibres, as it can be seen during each spatic action of the orbicularis to move downwards and become wrinkled. The tarsus, on the other hand, is too fixed to follow the action of the muscle. Now the cellular tissue and integument of the lid will, by this oft repeated blepharospasm, become loosened from the cartilage beneath and hang in a fold over its free border.

This displacement or dropping down of the integument and orbicularis fibres then, is the direct and common cause of entropion. With these changes the ciliæ are naturally displaced, and their direction changed—trichiasis.

Opeaators have heretofore thought to correct this condition by simply excising an elliptical fold of integument from the lid; in other words, they excised what they considered the superfluous skin, hoping by this shortening of the integument to render the traction sufficient to pull back the inverted lid to its normal position.

The explanation of the frequent failures of this operation is that it gains no fixed point of traction, and the loose integument at the upper part of the orbit and just beneath the brow immediately slips down to fill up the gap made by the wound; especially does this take place in old persons whose skin is flabby and loose, and in many cases the operation has been performed as many as four times on the same person without succeeding in gaining a fixed point of traction sufficient to overcome the entropion.

Another and still older operation for the relief of the trichiasis that follows entropion, was to split the free border of the lid internal to the ciliæ, and by a counter incision on the outside remove a quadrangular piece of the lid border containing the roots of the ciliæ; this, of course, removed the irritation produced by the eyelashes rubbing on the ball and allowed the cornea to regain its transparency, but it left a bald eye, which was ever afterwards an unsightly object.

I will now describe the operation as first performed by Hotz, and I do not think I can better do it than by using a part of his language:

"After the patient has been etherized an assistant fixes the skin of the brow against the upper orbital margin, the centre of the free edge of the lid is then seized with a pair of forceps and drawn downwards till the skin is moderately stretched. While the lid is held in this position, the point of a scalpel is applied 2 m.m. over the inner canthus and drawn across the lid in a horizontal line to a point 2 m.m. above the external canthus.

"As soon as the integument is cut the incision is changed into a gaping wound by the reaction of its upper border. Sometimes the muscular layer is cut with the skin and the cartilage at once; if not, the orbicularis is divided by a few careful strokes of the knife, keeping close to the lower border of the incision.

'The forceps which have been used for the purpose of drawing the lid downwards are now taken off to allow the lid to recover its natural position.

"The assistant applying forceps to the middle of the lower border of the wound, everts the skin to expose the muscle, and with a pair of fine forceps the muscular fibres next to the border of the skin are grasped and dissected off to the extent of a strip 3 or 4 m.m in width all along the border from canthus to canthus."

In making the sutures fine black silk is preferable, because its color is innocuous and it can be easier seen in the removal of the stitches.

Four or five sutures are usually requisite for a nice and perfect closure of the wound. The cutaneous borders are nicely approximated, and together drawn firmly down to the upper border of the cartilage, by first making a surgical knot and then pushing it down with both fingers in the same manner in which we close the ligature of a deeply situated artery.

The treatment after the operation is very simple; cold water compresses are grateful to the patient, and useful in so far as they limit the reactive swelling. After 24 or 36 hours, all dressings may be left off with directions to keep the eye clean. The sutures should be removed on the third day, when the wound will generally be found to be healed by first intention.

Since I saw this operation first performed in New York, now nearly two years ago, I have made it seven times, and in all of the seven with the best success. Three were upon the upper lid, and two upon the lower, and two upon both the upper and lower lids. In the first one of these latter cases, where there was an entropion and trichiasis of both upper and lower lids, I made the Hotz operation upon both lids at one sitting, but the inflammation and œdema that followed was so violent that I determined in the next case to make the operations separate. A few weeks afterwards I operated upon the other case in which both lids were affected, but upon one lid at a time, and thus avoided a great part of the inflammatory reaction that was the result in the first. The final result in both cases, however, was good.

Case third was in a young woman who had suffered for ten years with trachoma which resulted in entropion and trichiasis. The lids were almost bald, most of the larger ciliæ having been epilated.

I made a Hotz operation upon both upper lids, and in three months she had long luxuriant lashes and the pannus had so much cleared up that her vision was increased from 20-C to 20-XL.

CASE 4—Was a partial trichiasis of only $\frac{1}{3}$ inch of the upper lid. The ciliæ in this space had been time and again epilated when he came to me for a radical cure. I made a single incision $\frac{1}{2}$ inch in length near the upper border of the tarsal cartilage dissected out a small part of the muscle; took one stitch to bind the integument of the lower side down to the cartilage, when the wound healed by first intenton and the lashes of the affected portion came into line with the others.

CASE 5—Was a typical case of complete inversion of the lower lid with the cilia rolled up in the lower cul-de-sae of the coujnnctiva—the patient had suffered from granular lids for twelve years. The old treatment of this case would have been either to excise a transverse fold of the integument of the lid or to insert through the base of a transverse fold two or three ligatures.

Tne most serious objection to either of these operations is that they depend for their success upon two conditions:—the contraction of cicatricial tissue and the state of the integument upon the cheek; that is to say, we would have to rely upon one unknown quantity, an X as mathematicians would express it, for we cannot know how much or how little the scar may shrink; we trust, therefore, the ultimate result of the operation to an agency which is entirely beyond our control, and consequently should not be surprised if all of our calculations, based upon an unknown quantity, X, should deceive us in its results. Again, if the integument of the cheek be loose, the permanent success is still more likely to be illusory than before.

In order to evert the border of the lid the integument has by the operation been shortened to produce the requisite traction, but to make this traction effective and successful, the opposite end of the contracting cicatricial band must be firmly and immovably fixed; if not, the traction will operate upon both ends and the greater the degree of mobility alone will decide which will be moved the most.

If the integument of the cheek be firm, it forms a tolerably good basis for the traction; but if it be flacid, as it is in old people, it will be seen that the skin will move upwards rather than the entropion will be overcome. Hence the advantage of Hotz's operation on the lower as well as the upper lid, in that it attaches the integument to the border of the tarsal cartilage and thus gives a solid basis for traction.

The next case did not differ in any material respect from case four, except that I made the operation in only one eye. But case *seven* was the most interesting of all, since it gave the most perfect results. Male, age about thirty, who had been suffering from entropion and trichiasis for about eighteen years, the cilia of both upper lids had an abnormal position in the lower cul-de-sac, and if the lids were sufficlently opened to pull them from this position, on closing them again a violent spasm of the orbicularis would follcw and continue till the lashes were replaced under the lower lid.

The result of this condition after so many years, was a denseopacity of not only the upper portion of the cornea, but throughout its whole extent.

On making a Hotz operation upon this patient, on account of the flabby condition of the integument of the lids, I made a conical incision and dissected out the intervening integument.

His vision was only 20-cc at the time of the operation, but in two months after the cilia had been lifted from the cornea his vision rose to 20-lxx, and with a 10 glass it rose to 20-xl, and I have no doubt that as the opacity of the cornea gradually clears up his vision, with a ten glass, will reach 20-xxx.

The wound healed kindly, leaving the ciliæ in a perfectly normal position, and only a small transverse cicatrix.

To briefly recapitulate the advantages of this operation over all others:

1. Less of the integument is sacrificed, and consequently the scar is less unsightly.

2. The possibility of a relapse after the operation is inappreciably small.

3. One operation is, as a rule, sufficient to correct the deformity, because the distal border of the cartilage gives a fixed point for traction.

4. The wound, as a rule, heals by first intention, whereas when so much integument is sacrificed suppuration is the rule.

5. Its superiority over all other operations is more marked in old persons with flabby and loose skin, because of the impossibility to otherwise gain a fixed point for traction in such cases.

